# Accuracy of dynamic sentinel lymph node biopsy for inguinal lymph node staging in cN0 penile cancer

**DOI:** 10.1186/s13550-023-01013-1

**Published:** 2023-06-23

**Authors:** Juanito Gebruers, Laura Elst, Marcella Baldewijns, Liesbeth De Wever, Koen Van Laere, Maarten Albersen, Karolien Goffin

**Affiliations:** 1 Nuclear Medicine, University Hospitals Leuven, Herestraat 49, 3000 Leuven, Belgium; 2 Urology, University Hospitals Leuven, Leuven, Belgium; 3 Pathology, University Hospitals Leuven, Leuven, Belgium; 4 Radiology, University Hospitals Leuven, Leuven, Belgium; 5 Nuclear Medicine and Molecular Imaging, Department of Imaging and Pathology, KU Leuven, Leuven, Belgium

**Keywords:** Penile cancer, Dynamic sentinel lymph node biopsy, SPECT/CT

## Abstract

**Background:**

Penile cancer is characterized by an early lymphatic dissemination. In intermediate and high-risk primary tumors without palpable inguinal lymph nodes, there is a 6–30% risk of micro-metastatic disease. Invasive lymph node staging in these patients is performed using dynamic sentinel lymph node biopsy (DSNB). In this study, the role of DSNB in cN0 penile cancer was studied, evaluating features of sentinel lymph node (SN) visualization and outcome parameters. Patients with penile cancer without inguinal lymph node metastases who were referred for DSNB at our center between January 2015 and May 2021 and had a follow-up period of at least 18 months, were retrospectively included. After injection of 85 ± 20 MBq [^99m^Tc]Tc-nanocolloid peritumorally, dynamic, static planar and SPECT/CT imaging was performed. Primary endpoints were sensitivity of the diagnostic procedure, disease-free survival and DSNB-related adverse events. Secondary endpoints were SN detection rate, number of SNs and the number of counts of the most active SN.

**Results:**

Seventy-seven penile DSNB procedures in 75 patients (67 ± 11 years) were included. The detection rate of DSNB was 91% and 96% per procedure and groin, respectively. Sensitivity, specificity, negative predictive value (NPV) and positive predictive value (PPV) were 79%, 100%, 97% and 100%, respectively. More SNs were seen on SPECT/CT than on static planar imaging (1.33 vs. 1.17, *p* = 0.001). The mean counts per SN on static planar imaging was lower compared to SPECT/CT (1343 vs. 5008; *p* < 0.0001). There was a positive correlation between the total counts of the SN on the static planar image and the SPECT/CT (*r* = 0.79, *p* < 0.0001). Only one out of seventy-five patients (1%) experienced DSNB-related adverse events. After 18 months, 58 patients remained disease free (77%), 13 developed local recurrence (17%), and 4 developed lymphatic or distant metastases (5%).

**Conclusion:**

DNSB is a safe diagnostic procedure with a good detection rate and in particular high negative predictive value. It can therefore prevent overtreatment of patients with negative inguinal groins on clinical examination and DSNB examination. Finally, DSNB enables an early detection of occult metastases which would not be visualized with standardized imaging modalities.

## Background

Penile cancer is a rare malignancy with an incidence of approximately 1/100,000 in Europe and the USA and higher incidences in other regions such as South America, Southeast Asia and Africa, where penile cancer accounts for 1–2% of all malignancies. Several pathological types are known with squamous cell carcinoma being the most common (95% of cases) [1]. The most important prognostic factor in survival is the presence and extent of nodal metastases, with 5-yr cancer specific survival (CSS) of approximately 95%, 80%, 65% and 35% for N0, N1, N2 and N3 disease, respectively [2]. As penile cancer is characterized by early lymphatic spread and conventional imaging is inadequate for the detection of micro-metastatic disease, accurate and upfront surgical staging of the inguinal lymph nodes is crucial in disease management [3]. Moreover, complications after radical inguinal lymphadenectomy (ILND), such as lymphocele formation, hematoma, wound infection, wound dehiscence and bleeding, can occur in up to 58% of patients, highlighting the importance of adequate primary staging to prevent overtreatment in patients without nodal metastases [4, 5].

The European Association of Urology (EAU) recommends a physical examination and recording of morphology, extent and invasion of penile structures for staging of the primary tumor. For clinical lymph node staging, a physical examination of both groins should be performed to assess the presence, number, laterality and characteristics of inguinal lymph nodes. In patients with clinical N0 (cN0) disease, the EAU guidelines recommend inguinal ultrasound (US) imaging and fine needle aspiration cytology (FNAC) before surgical staging by DSNB [1]. A positive ultrasound and FNAC reduces the need for dynamic sentinel lymph node biopsy (DSNB) and enables earlier curative ILND. Unfortunately, a negative ultrasound cannot exclude the presence of micro-metastatic disease and the risk of occult metastases in inguinal lymph nodes is 6–30% [6, 7].

Therefore, DSNB is advocated to be performed in intermediate and high-risk cN0 patients, defined as non-palpable, non-suspicious on inguinal ultrasound and with negative FNAC results [1]. The urologist Ramon Cabañas was the first to describe the sentinel node (SN) concept for patients with penile cancer in 1977, where he defined the SN as the first lymph node on a direct drainage pathway from the primary tumor [8]. In DSNB, three to four deposits of a radioactive tracer are injected circumferentially peritumorally. Tracer injection is followed by dynamic lymphoscintigraphy, as well as static planar and SPECT/CT imaging to localize the lymph trajectory and the SN [7, 9]. Per-operatively, the SN can be located by a gamma probe for resection. In the current study, we evaluated the role of DSNB for cN0 penile cancer management, evaluating on the one hand the determinant features for SN visualization and identification, and on the other hand outcome parameters.

## Methods

### Patient selection and clinical staging

In this retrospective single-center study, patients with intermediate (pT1aG2) or high-risk (≥ pT1b) penile carcinoma (primary or local recurrence) in whom a DSNB was performed at our center between January 2015 and May 2021 were included. All patients had a follow-up period of at least 18 months. In addition to routine clinical examination, patients underwent inguinal US imaging and in case of suspicious lymph nodes additional core needle biopsy was performed. Patients who showed affected lymph nodes on core needle biopsy were scheduled for an ipsilateral ILND, and in case of negative lymph nodes on clinical examination, US and/or core needle biopsy, additional DSNB was performed. Patients with a history of DSNB or inguinal lymph node resection were excluded. Also patients with evidence of distant metastases were excluded. The following clinical parameters were obtained from the patient records at baseline (preoperatively): medical history, primary staging, body mass index (BMI), American Society of Anesthesiologists (ASA) score and Eastern Cooperative Oncology Group (ECOG) score. Age at initial diagnosis was reported according to the first available pathological report positive for penile carcinoma.

### DSNB procedure

Using a one-day protocol, an average activity of 85 ± 20 MBq [^99m^Tc]Tc-nanocolloid in a volume of 0.3 ml was injected peritumorally (3–4 deposits). A dual-head SPECT/CT gammacamera with medium-energy low-penetration (MELP) collimators was used (Symbia T series, Siemens Healthineers, Erlangen, Germany). First a 15 min dynamic recording was started immediately following tracer injection (90 frames of 10 s, matrix 128 × 128), with the detector positioned anteriorly at the level of the pelvis. This procedure was repeated until a SN was visible (uni- or bilaterally) up to a maximum of 60 min. An anterior static planar image of 5 min was acquired afterward (matrix 128 × 128). Finally, a SPECT/CT of the pelvis was performed to detect and localize the SNs (60 views, 8 s per view, matrix 128 × 128). A vendor-based iterative reconstruction using ordered-subset expectation maximization algorithms with 15 iterations and 6 subsets was performed (“Flash3D”) with low-dose CT-based attenuation correction scatter correction. Late imaging (static planar and/or SPECT/CT) was acquired, with or without additional tracer injection, if no SN was visible after the initial imaging phase. Using a cobalt source, SNs were marked on the skin. Peri-operatively, the SNs were detected by the surgeon using a rigid gamma probe and removed. In addition, after administration of blue dye per-operatively, all blue lymph nodes, as well as palpable enlarged lymph nodes were resected. Finally, the gamma probe was used to measure the background activity in the groin to ensure that no radioactive lymph nodes had been missed.

### Image analysis

Visual and semi-quantitative analysis was performed using MIM^®^ (v7.0; MIM software Inc., Cleveland, OH, USA). First, the total duration of the dynamic acquisition was reported since this represents the time to visualization of the SN. Second, the static planar images were reviewed and the number of SNs in each inguinal region was determined. Additionally, the counts of the SN with the highest tracer uptake were recorded. Then, the same data were derived for the SPECT/CT images. Finally, the number of SNs on static vs. SPECT/CT was compared and in case of discrepancy, the rationale for discrepancy was reported.

### Pathology and follow-up

Per SN resection procedure, the number of nodes removed per groin, the number of affected nodes and the presence of capsular extension were noted. For additional lymphadenectomies that were performed in case of histologically positive SNs, the number of resected nodes, the number of affected nodes and the presence of capsular extension were noted. Finally, we reported the complications due to DSNB procedure according to the Clavien–Dindo classification system [10]. In addition, the occurrence of recurrence (local, inguinal, pelvic, distant) was noted and the time interval from the date of DSNB was reported.

### Statistical analysis

The number of identified SNs between imaging modalities was compared using a paired *t* test. Disease-free survival after negative DSNB was estimated using a Kaplan–Meier method. Pearson correlation analysis was performed on the counts of the SN with the highest tracer uptake on static planar versus SPECT/CT images. Statistical analyses were performed using GraphPad Prism (v 9.5.1, Dotmatics, San Diego, California, USA). To determine sensitivity, specificity, NPV and PPV, the following criteria were defined: 1/a false negative finding is a negative SN on pathology with an inguinal recurrence occurring within 18 months after DSNB; 2/a true positive finding is a SN that was positive on pathology and for which the patient additionally received a lymphadenectomy; 3/a true negative finding is a negative SN on pathology and the groin remained negative within the 18 months of follow-up; and 4/a false positive result cannot be present since a histologic confirmation of cancer spread to groin lymph node(s) at DSNB cannot be falsified [11].

## Results

### Patient characteristics

In this retrospective study, 77 penile DSNB procedures in 75 patients were included (mean age at diagnosis 67 ± 11 years; Table 1). The procedure was repeated unilaterally in 2 patients because of initial non-visualization of the SN. Histopathological T stage was T1a in 14 patients, T1b in 16 patients, T2 in 37 patients and T3 in 8 patients. The most common histologic subtype was common squamous cell carcinoma in 23 patients, followed by mixed carcinoma in 13 patients, basaloid carcinoma in 5 patients, verrucous carcinoma in 4 patients, warty basaloid carcinoma in 2 patients, papillary carcinoma in 1 patient and sarcomatoid carcinoma in 1 patient. In 26 patients, the subtype was not determined (Table 1). In the available procedures, 12 groins were excluded since unilateral ILND was already planned because of clinically suspected inguinal lymph nodes (9 groins) or positive lymph node after US imaging + core needle biopsy (3 groins). Using these parameters, 138 groins were available for DSNB procedure evaluation (71 right, 67 left).

**Table 1:** Patients characteristics and clinical parameters

Total number of patients	75
Mean age at diagnosis	67 ± 11 years old
ASA score	
1	9
2	46
3	19
4	1
BMI (mean)	28
Indication	
Primary diagnosis	63
Recurrence	12
Time between first diagnosis and DSNB	
Primary diagnosis: mean and median	46 and 39 days
Recurrence: mean and median	1030 and 514 days

*ASA score*, American Society of Anesthesiologists, *BMI*, body mass index

### Imaging characteristics

**Fig. 1:**
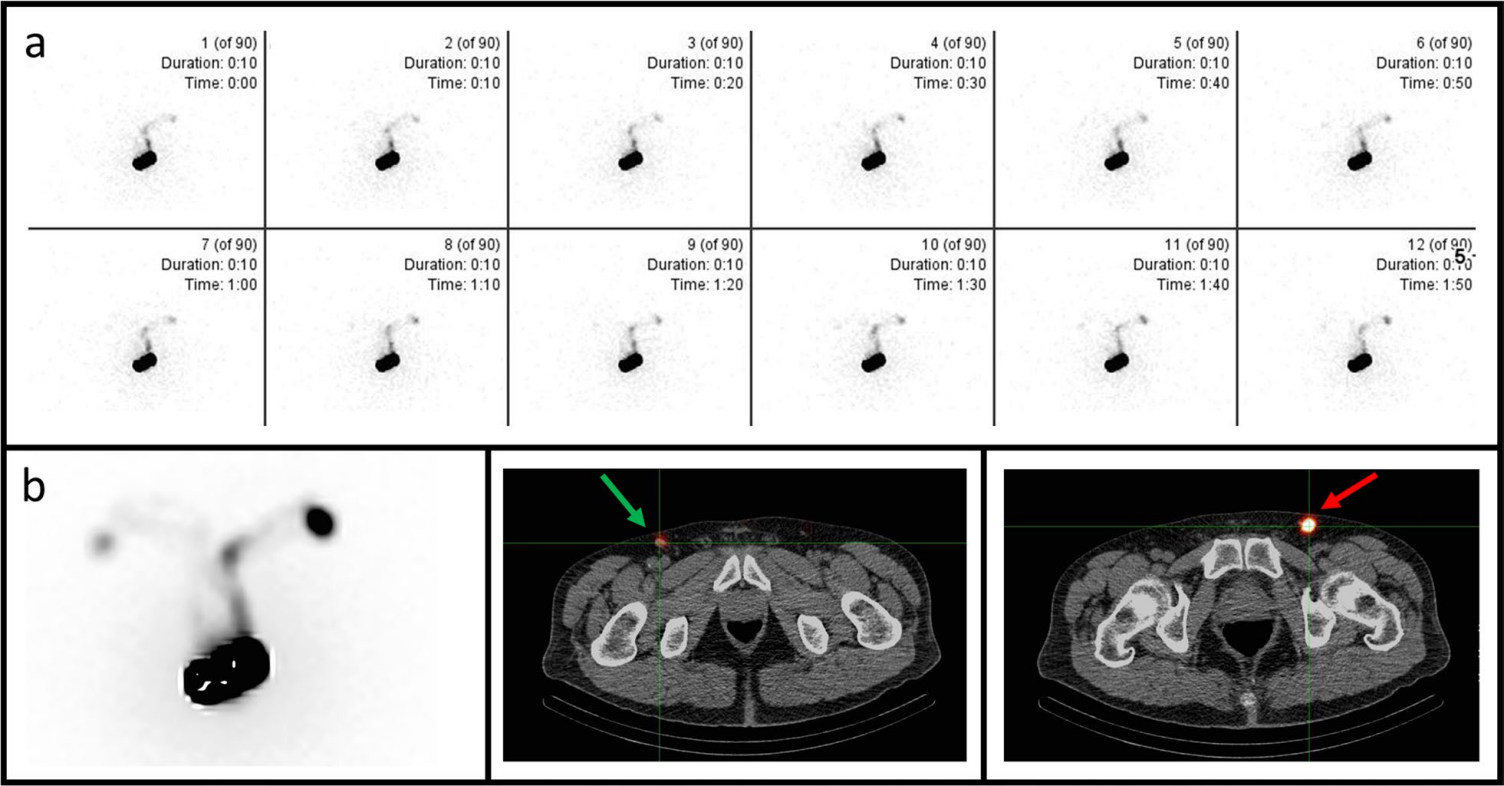
Example of a patient with bilateral detection of the SN in both inguinal groins. **a**: dynamic imaging showing the appearance of the SN first on the left inguinal region (0:10 min) and afterward on the right side (1:20 min). **b**: static planar image showing the inguinal SN on both sides (left and right). SPECT/CT showing the localization of the inguinal SN on the left (red arrow) and right (green arrow) side

In 75% of evaluated procedures, a SN was observed (uni- or bilaterally depending on the indication) after an average of 22 min (range 15–60 min) (Fig. 1). A total of 19 (25%) procedures received late imaging due to non-visualization on the first image phase, this after an additional injection of an average of 85 ± 25 MBq [^99m^Tc]Tc-nanocolloid in 16 out of 19 procedures (84%).

DSNB procedure was successful, i.e., a SN could be identified bilaterally or unilaterally, depending on the indication, in 70 procedures (91%). In 2 patients with unilateral non-visualization, the DSNB procedure was repeated unilaterally and showed a SN. Finally, in 5 groins no SN could be identified (detection rate per groin 96%).

**Fig. 2:**
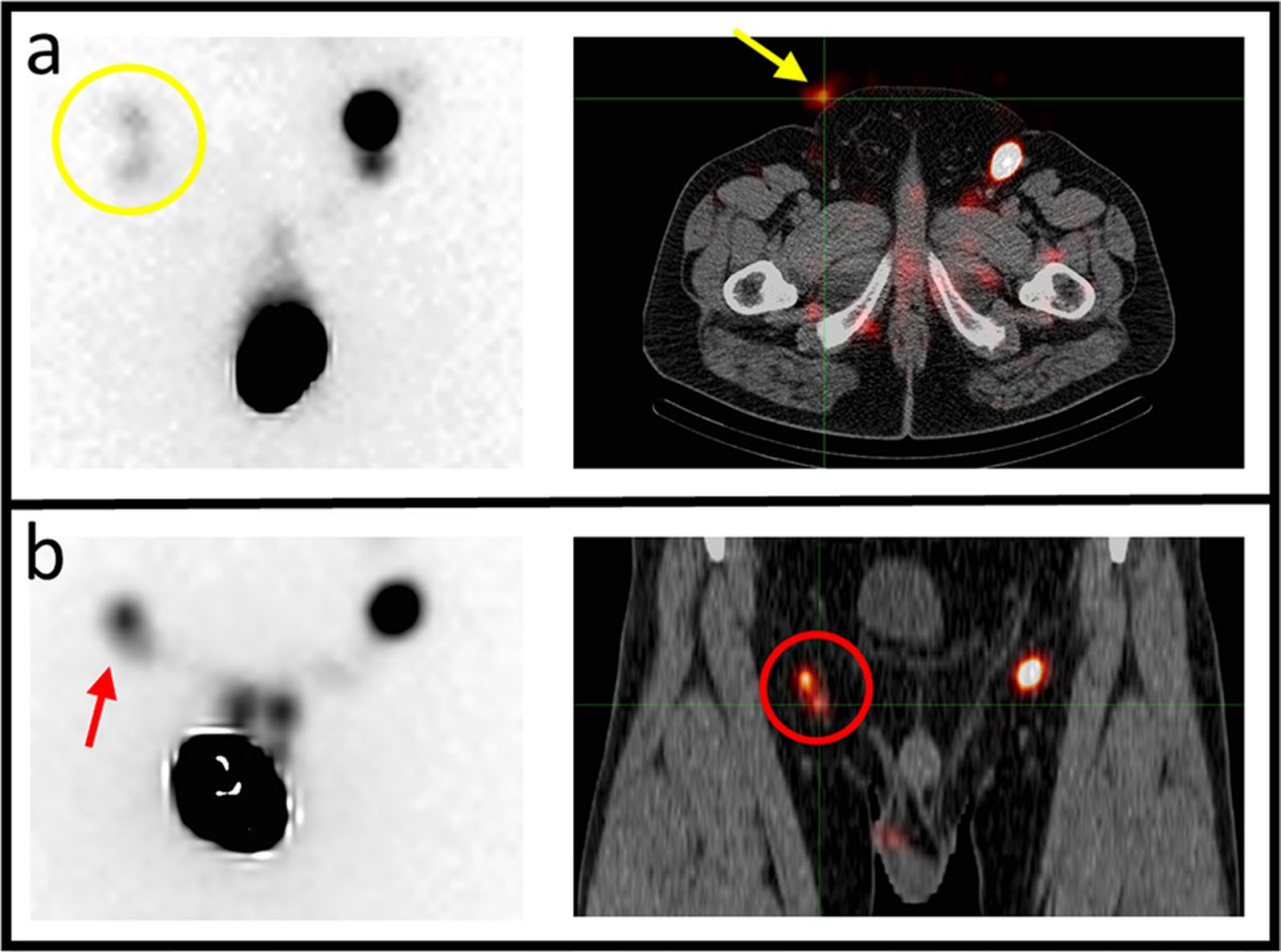
Discrepant findings between static planar and SPECT/CT imaging. **a**: static planar (yellow circle) > SPECT/CT image, falsely higher due to contamination on the skin (yellow arrow). **b**: static planar < SPECT/CT image (red circle), due to superposition (red arrow) of two SNs on the static planar image

**Fig. 3:**
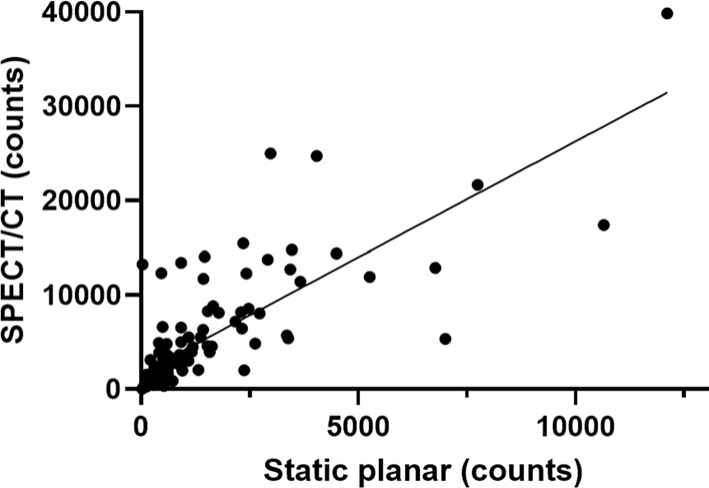
Correlation of the counts of the SN with the highest tracer uptake on static planar versus SPECT/CT imaging. The counts on SPECT/CT were significantly higher; however, the comparison shows a good correlation (black line)

The use of SPECT/CT resulted in the identification of a significantly higher number of SNs compared to static planar imaging (1.33 vs. 1.17, *p* = 0.001). This was mainly due to superposition of the SNs on the static image (example in Fig. 2). In 2 groins, there was no visualization of a SN on the static planar image with visualization of one SN on the SPECT/CT. Inversely, in some cases a perceived higher total number of SNs were seen on static planar vs. SPECT/CT images, due to tracer contamination on the skin or lymphatic vessel stasis (example in Fig. 2). In 1 groin, a SN was visualized on the static planar image without confirmation on the SPECT/CT (non-visualization); this patient did not undergo a DSNB and was planned for a modified ILND. The counts in the SN with the highest tracer uptake were significantly higher on SPECT/CT than on static planar imaging (5008 vs. 1343, *p* < 0.001). Moreover, the counts in the SN with the highest tracer uptake on SPECT/CT and static planar imaging were significantly correlated (*r* = 0.79, *p* < 0.0001, Fig. 3). The number of actual resected lymph nodes (LN) in turn was significantly higher than the number of SNs identified on SPECT/CT (1.73 vs. 1.43, *p* < 0.0001) (Table 2).

**Table 2:** Imaging characteristics

Total number of procedures	77
Time to visualization of SN (mean)	22 min (range 15–60 min)
Late imaging	19 procedures
Additional tracer injection	16 procedures
Detection rate	
Per procedure	91%
Per groin	96%

Comparison imaging	Planar static imaging	SPECT/CT imaging
Number of SNs (mean)	1.17	1.33
Count of most active SN (mean)	1343	5008

Comparison resection	SPECT/CT imaging	Resection
Number of SNs (mean)	1.43	1.73

### Follow-up

In 1 patient, after DSNB an abscess occurred in the groin requiring drainage (grade III). No complications were reported in the other seventy-four patients.

In 5 out of 138 groins (4%), no SN could be identified. In 1 groin with non-visualization, a modified ILND was performed which showed 12 negative LNs and the patient remained disease free during follow-up. In the other 4 groins (4 patients), no LAD was performed and these groins remained disease free during follow-up. In these four patients, the primary tumor was intermediate in one and high risk in three, respectively.

**Fig. 4:**
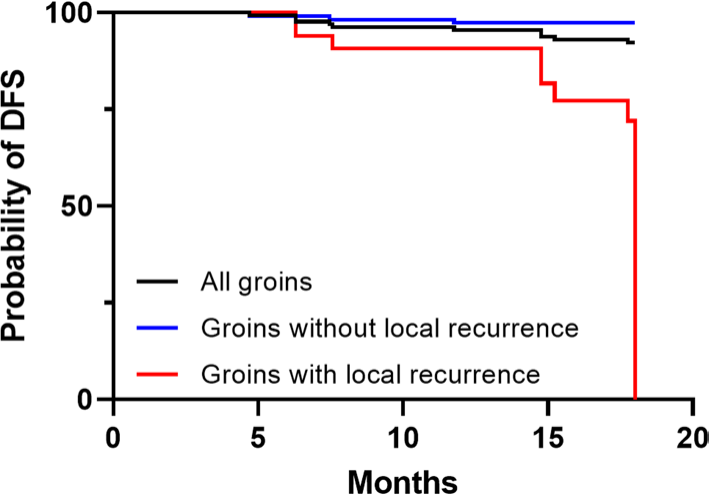
Kaplan–Meier representation of DFS per groin

During the follow-up period of 18 months after successful DSNB, 24 groins (13 patients) were excluded from analysis considering the occurrence of local recurrence (on average 8.8 months after DSNB). These groins were excluded since the procedure was not representative anymore for the initial malignancy due to the recurrence. Three groins (2 patients) developed inguinal recurrence: this occurred after 4.7 and 11.8 months in one patient (bilateral nodal inguinal recurrence) and after 7.4 months in the other patient. These three groins were considered as false negative (3%). Ninety-five groins remained negative in the follow-up period (87%) and 11 groins true positive (10%), resulting in a sensitivity of 79%, specificity of 100%, NPV of 97% and PPV of 100% (Fig. 4). Taking these parameters into account, the false negative rate (FNR) was 21%.

Of the 11 groins with a positive SN (3 with capsular extension) on pathology, additional ILND was performed in 9 groins (9 patients). One of these nine ILNDs showed additional inguinal nodal involvement, however, without capsular extension. Of the 2 groins (2 patients) who did not receive additional ILND on individual basis, 1 remained disease free (isolated tumor cells in the SN) and the other developed hematogenous metastases after 12.6 months without inguinal recurrence.

## Discussion

This study assessed the performance and safety of DSNB in patients with penile cancer and cN0 inguinal stage per groin. Primary endpoints were sensitivity of the procedure, disease-free survival (NPV) and DSNB-related adverse events. Secondary scan-related endpoints were visualization of a SN (detection rate), number of SNs and the number of counts at the most active SN.

The detection rate in this study was 91% and 96% per procedure and groin, respectively, and is therefore generally in line with available literature. Wever et al. reported a detection rate of 92% in 644 patients and Sedigh et al. reported a detection rate of 80% in 35 patients [9, 12]. Valdés Olmos et al. reported a detection rate of 97% in their patient cohort; however, unilateral visualization of the SN was considered a successful procedure, which was not the case in this study [13]. In patients with non-visualization, repetition of the DSNB procedure could be an option, as was done in two patients in this study. In these patients, a SN could be identified on the repeat DSNB. Likewise, Sahdev and colleagues identified 20 out of 166 patients with unilateral non-visualization of the SN. Of these 20 patients, 7 underwent repeat DSNB at a later date, with 6 having successful visualization of the SN [14].

In this study, the number of SNs on SPECT/CT was significantly higher than on static planar imaging. SPECT/CT provides 3-dimensional data facilitating the evaluation of the groin and the underlying SNs.

Moreover, due to the lower image contrast, SNs with limited tracer uptake above the superimposed background cannot be visualized by static planar imaging and only by SPECT/CT. This study in one of the largest patient cohorts with penile cancer showed that SPECT/CT outperforms static planar imaging in detecting SNs per groin. This is in analogy to the study of Saad et al., in which the addition of SPECT/CT proved to be significant in 76% of their patient cohort. SPECT/CT provided superior images with increased nodal yield, more precise localization, a clearer distribution and drainage pattern, and a significant reduction in false extranodal hot spots observed on conventional static planar imaging [15]. A previous study by Jimenez-Hefferman et al. also evaluated this comparison in a large group of 1508 patients, yet only seven patients of this cohort had penile carcinoma. In their study, SPECT/CT also had an influence on the drainage territory of the malignancy; however, this was not the case in this study, since the penis always drains to the inguinal nodes [16]. In 1 groin, a SN was suspected on the static planar image without confirmation on the SPECT/CT (non-visualization). In this patient, a modified ILND was performed which showed 12 negative LNs and the patient remained disease-free during follow-up. Possibly, the focus of increased tracer uptake on the planar static image could represent stasis of tracer in a lymphatic vessel.

In turn, the number of resected lymph nodes was significantly higher than the number of SNs on SPECT/CT. This discrepancy is likely a result of the surgical procedure, which uses besides a gamma probe, also blue dye and palpation of the groin to find SNs.

When analyzing the counts of the SN with the highest uptake, a significantly higher value was obtained for SPECT/CT compared to static planar imaging. Since both imaging modalities are acquired immediately one after the other, it seems unlikely that more tracer would have entered the SNs by the time of the SPECT/CT as explanation of this phenomenon. However, static planar images are not corrected for body attenuation and SPECT/CT has a higher signal-to-noise ratio. Furthermore, no geometric mean could be calculated to correct for this phenomenon on the static planar images given that only anterior acquisitions were available. More data are needed regarding the correlation of SN characteristics on SPECT/CT and their associated pathologically proven positivity. Currently, no data are available about the counts of SNs on SPECT/CT and their associated tumor load.

In this manuscript, the dynamic images were not included in the semi-quantitative analysis of the SN. These dynamic images are mainly important to objectify the visualization of the SN. Additional static planar and SPECT/CT imaging are subsequently performed to localize the SNs. The duration of the dynamic acquisition represents the time to visualization after injection of the radioactive tracer. In this study, a SN could be seen after an average of 22 min. An alternative procedure workflow could be to inject the patient at the nuclear medicine department and acquire a static planar image 60 min after injection. If SNs are visualized, additional SPECT/CT can be performed, if not, additional injection and late imaging is required.

In our study, DSNB had a sensitivity of 79% and three false negative groins were reported. An explanation for the false negative results could be that the SN was not detected due to malignant invasion, possibly due to tumor blockage limiting tracer uptake [17]. Besides hematoxylin and eosin staining, immunohistochemistry should be performed in the evaluation of the SN, since it can detect micro-metastatic disease smaller than 2 mm [11]. Using DSNB, Valdés Olmos et al. reported a sensitivity of 89% for detection of inguinal lymph node metastases in 74 patients with penile cancer [13]. Hadway et al. showed a sensitivity of 95% and FNR of 5%; however, this was in a follow-up period of 11 months [18]. A recent systematic review and meta-analysis published in 2022 showed a pooled sensitivity of 87% (82–91) in 2893 patients (28 studies) [11]. In another systematic review and meta-analysis by Zou et al., a pooled sensitivity and NPV of 88% (95% CI 84–90%) and 99% (98–99%), respectively, were found [19]. In a large prospective study in 264 patients (500 groins), a sensitivity of 92% and 95% was found for the DSNB alone and with ultrasound, respectively [20]. The sensitivity in our study is lower in comparison with these data, which may be attributed to the fact that 12 groins were excluded as a result of positive US and pre-DSNB (core needle) biopsy, leading to a low number of true positives compared with earlier studies. Besides, two true positives of the DSNB were excluded from the analysis as a result of local recurrence. Last, this series includes data from the initiation of the use of DSNB in penile cancer and a learning curve effect may contribute to low sensitivity compared to high volume centers with established extensive experience.

In our study, the FNR was 21%, which is higher than reported in a previously published study by Dimopoulos et al. (5.1%) in 151 patients, a study in 264 patients by Lam et al. (5%) and a study by Leijte et al. (7%) in 323 patients [20–22]. One of the landmark studies on this topic published in 2005 by Kroon et al. showed a FNR of 16% [23].

The complication rate of surgery in our study was low (1% of patients), which is much lower than the complication rate in the study by Dimopoulos et al. (complication rate 21%) and more in line with the results of Leitje et al. (complication rate 4.7%) [21, 22]. However, the study by Dimopoulos recorded their complications prospectively and in their study, the majority (10.7%) of complications were Grade I, due to groin swelling requiring no intervention [21]. Our retrospective study is conducted within a concentrated care model and likely underestimates the total number and grade of complications as patients may have presented elsewhere with complications not captured in our records.

There are some limitations to this study. This study was retrospective and future prospective studies could provide more insights on the performance of DSNB in penile cancer. In this study, a period of 18 months was used as a reliable interval for a SN. However, a recent systematic review showed that a 12-month period also shows reliable ground truth [11]. If this is considered for this study, it would not affect the false negative results, but it would affect the true negatives given that four local recurrences could then be excluded, achieving eight supplementary true negative groins and also a higher NPV. Moreover, the sensitivity and FNR were calculated taking into account the information of only the DSNB procedure and not of the entire diagnostic work up (clinical examination + US + core needle biopsy + DNSB). As a result, there is an underestimation of the sensitivity and overestimation of the FNR since 12 groins were already initially scheduled for an ILND and thus 12 additional true positives were not taken into account. Secondly, a very widely geographical patient group were referred to our center due to its expertise in the topic and some interesting information was therefore not available in the medical report: (1) the type of surgery on the primary tumor, possibly explaining the number of local recurrences; (2) follow-up of complications, possibly resulting in an underestimation of complication rate. Counts of the SN reported by the gamma probe per-operatively were not included in this study, since a longer period of time elapsed between SPECT/CT acquisition and resection, so comparison of the amount of counts would be irrelevant since more tracer could have entered the SNs. Also, exact comparison of individual SNs between SPECT/CT and resection is not feasible.

## Conclusion

DNSB is a safe diagnostic procedure with a good detection rate and in particular high negative predictive value. It can therefore prevent overtreatment of patients with negative inguinal groins on clinical examination and DSNB examination. Finally, DSNB enables an early detection of occult metastases which would not be visualized with standardized imaging modalities.

## Data Availability

Pseudonymized data will be deposited in an access-controlled file server used by the academic research imaging group UZ Leuven/KU Leuven, which will be shared on reasonable request from a qualified investigator on approval by the Ethics Committee of the local university hospital.

## Abbreviations

ASA: American society of anesthesiologists
BMI: Body mass index
CI: Confidence interval
cN0: Clinical node negative stage
CT: Computed tomography
DSNB: Dynamic sentinel lymph node biopsy
ECOG: Eastern cooperative oncology group
FDG PET/CT: [^18^F]fluorodeoxyglucose positron emission tomography
FNAC: Fine needle aspiration
FNR: False negative rate
ILND: Inguinal lymphadenectomy
LN: Lymph node
MBq: Megabecquerel
MELP: Medium-energy low-penetration
NPV: Negative predictive value
PPV: Positive predictive value
SN: Sentinel lymph node
SPECT: Single-photon emission computed tomography
TNM: Tumor, node and metastasis
US: Ultrasound imaging

## References

[1] Brouwer OR, Albersen M, Parnham A, Protzel C, Pettaway CA, Ayres B, Antunes-Lopes T, Barreto L, Campi R, Crook J, Fernández-Pello S, Greco I, van der Heijden MS, Johnstone PAS, Kailavasan M, Manzie K, Marcus JD, Necchi A, Oliveira P, Osborne J, Pagliaro LC, Garcia-Perdomo HA, Rumble RB, Sachdeva A, Sakalis VI, Zapala Ł, Sánchez Martínez DF, Spiess PE, Tagawa ST (2023). European association of urology-american society of clinical oncology collaborative guideline on penile cancer: 2023 update. Eur Urol.

[2] Horenblas S, Van Tinteren H (1994). Squamous cell carcinoma of the penis. IV. Prognostic factors of survival: analysis of tumor, nodes and metastasis classification system. J Urol.

[3] Thomas A, Necchi A, Muneer A, Tobias-Machado M, Tran ATH, Van Rompuy AS, Spiess PE, Albersen M (2021). Penile cancer. Nat Rev Dis Primers.

[4] Wever L, de Vries HM, Dell'Oglio P, van der Poel HG, Donswijk ML, Sikorska K, van Leeuwen FWB, Horenblas S, Brouwer OR (2022). Incidence and risk factor analysis of complications after sentinel node biopsy for penile cancer. BJU Int.

[5] Stuiver MM, Djajadiningrat RS, Graafland NM, Vincent AD, Lucas C, Horenblas S (2013). Early wound complications after inguinal lymphadenectomy in penile cancer: a historical cohort study and risk-factor analysis. Eur Urol.

[6] Fankhauser CD, de Vries HM, Roussel E, Jakobsen JK, Issa A, Lee EWC, Schifano N, Alnajjar H, Castiglione F, Antonelli L, Oliveira P, Lau M, Parnham A, Albersen M, Watkin NA, Muneer A, Ayres BE, Brouwer OR, Sangar V (2022). Lymphovascular and perineural invasion are risk factors for inguinal lymph node metastases in men with T1G2 penile cancer. J Cancer Res Clin Oncol.

[7] Ficarra V, Akduman B, Bouchot O, Palou J, Tobias-Machado M (2010). Prognostic factors in penile cancer. Urology.

[8] Cabanas RM (1977). An approach for the treatment of penile carcinoma. Cancer.

[9] Wever L, de Vries HM, van der Poel H, van Leeuwen F, Horenblas S, Brouwer O (2022). Minimally invasive evaluation of the clinically negative inguinal node in penile cancer: dynamic sentinel node biopsy. Urol Oncol.

[10] Clavien PA, Barkun J, de Oliveira ML, Vauthey JN, Dindo D, Schulick RD, de Santibañes E, Pekolj J, Slankamenac K, Bassi C, Graf R, Vonlanthen R, Padbury R, Cameron JL, Makuuchi M (2009). The Clavien-Dindo classification of surgical complications: five-year experience. Ann Surg.

[11] Fallara G, Pozzi E, Onur Cakir O, Tandogdu Z, Castiglione F, Salonia A, Alnajjar HM, Muneer A; EAU-YAU Penile and Testis Cancer Working Group. Diagnostic accuracy of dynamic sentinel lymph node biopsy for penile cancer: a systematic review and meta-analysis. Eur Urol Focus. 2022;S2405–4569(22):00277–2. 10.1016/j.euf.2022.11.018 36470729

[12] Sedigh O, Preto M, Soleimanzadeh F, Marra G, Falcone M, Rolle L, Ceruti C, Timpano M, Sibona M, Dalmasso E, Delmonte S, Caliendo V, Frea B, Gontero P (2018). Role of perioperative dynamic sentinel node biopsy for cN0 penile cancer management: experience from an Italian tertiary referral center. Tumori.

[13] Valdés Olmos RA, Tanis PJ, Hoefnagel CA, Jansen L, Nieweg OE, Meinhardt W, Horenblas S (2001). Penile lymphoscintigraphy for sentinel node identification. Eur J Nucl Med.

[14] Sahdev V, Albersen M, Christodoulidou M, Parnham A, Malone P, Nigam R, Bomanji J, Muneer A (2017). Management of non-visualization following dynamic sentinel lymph node biopsy for squamous cell carcinoma of the penis. BJU Int.

[15] Saad ZZ, Omorphos S, Michopoulou S, Gacinovic S, Malone P, Nigam R, Muneer A, Bomanji J (2017). Investigating the role of SPECT/CT in dynamic sentinel lymph node biopsy for penile cancers. Eur J Nucl Med Mol Imaging.

[16] Jimenez-Heffernan A, Ellmann A, Sado H, Huić D, Bal C, Parameswaran R, Giammarile F, Pruzzo R, Kostadinova I, Vorster M, Almeida P, Santiago J, Gambhir S, Sergieva S, Calderon A, Young GO, Valdes-Olmos R, Zaknun J, Magboo VP, Pascual TN (2015). Results of a prospective multicenter international atomic energy agency sentinel node trial on the value of SPECT/CT over planar imaging in various malignancies. J Nucl Med.

[17] Leijte JA, Kroon BK, Valdes Olmos RA, Nieweg OE, Horenblas S (2007). Reliability and safety of current dynamic sentinel node biopsy for penile carcinoma. Eur Urol.

[18] Hadway P, Smith Y, Corbishley C, Heenan S, Watkin NA (2007). Evaluation of dynamic lymphoscintigraphy and sentinel lymph-node biopsy for detecting occult metastases in patients with penile squamous cell carcinoma. BJU Int.

[19] Zou ZJ, Liu ZH, Tang LY, Wang YJ, Liang JY, Zhang RC, Tang YQ, Lu YP (2016). Radiocolloid-based dynamic sentinel lymph node biopsy in penile cancer with clinically negative inguinal lymph node: an updated systematic review and meta-analysis. Int Urol Nephrol.

[20] Lam W, Alnajjar HM, La-Touche S, Perry M, Sharma D, Corbishley C, Pilcher J, Heenan S, Watkin N (2013). Dynamic sentinel lymph node biopsy in patients with invasive squamous cell carcinoma of the penis: a prospective study of the long-term outcome of 500 inguinal basins assessed at a single institution. Eur Urol.

[21] Dimopoulos P, Christopoulos P, Shilito S, Gall Z, Murby B, Ashworth D, Taylor B, Carrington B, Shanks J, Clarke N, Ramani V, Parr N, Lau M, Sangar V (2016). Dynamic sentinel lymph node biopsy for penile cancer: a comparison between 1- and 2-day protocols. BJU Int.

[22] Leijte JA, Hughes B, Graafland NM, Kroon BK, Olmos RA, Nieweg OE, Corbishley C, Heenan S, Watkin N, Horenblas S (2009). Two-center evaluation of dynamic sentinel node biopsy for squamous cell carcinoma of the penis. J Clin Oncol.

[23] Kroon BK, Horenblas S, Meinhardt W, van der Poel HG, Bex A, van Tinteren H, Valdés Olmos RA, Nieweg OE (2005). Dynamic sentinel node biopsy in penile carcinoma: evaluation of 10 years experience. Eur Urol.

